# Surface Chemistry Dependence on Aluminum Doping in Ni-rich LiNi_0.8_Co_0.2−*y*_Al_*y*_O_2_ Cathodes

**DOI:** 10.1038/s41598-019-53932-6

**Published:** 2019-11-27

**Authors:** Zachary W. Lebens-Higgins, David M. Halat, Nicholas V. Faenza, Matthew J. Wahila, Manfred Mascheck, Tomas Wiell, Susanna K. Eriksson, Paul Palmgren, Jose Rodriguez, Fadwa Badway, Nathalie Pereira, Glenn G. Amatucci, Tien-Lin Lee, Clare P. Grey, Louis F. J. Piper

**Affiliations:** 1 Department of Physics, Applied Physics and Astronomy, Binghamton University, Binghamton, NY 13902 USA; 2 Department of Chemistry, University of Cambridge, Lensfield Road, Cambridge, CB2 1EW UK; 3 Present Address: Department of Chemical and Biomolecular Engineering, University of California, Berkeley, CA 94720 United States; 4 Energy Storage Research Group, Department of Materials Science and Engineering, Rutgers University, North Brunswick, NJ 08902 United States; 5 Scienta Omicron AB, P.O. Box 15120, 750 15 Uppsala, Sweden; 6 Diamond Light Source Ltd., Diamond House, Harwell Science and Innovation Campus, Didcot, Oxfordshire OX11 0DE UK; 7 Materials Science & Engineering, Binghamton University, Binghamton, NY 13902 USA

**Keywords:** Batteries, Characterization and analytical techniques

## Abstract

Aluminum is a common dopant across oxide cathodes for improving the bulk and cathode-electrolyte interface (CEI) stability. Aluminum in the bulk is known to enhance structural and thermal stability, yet the exact influence of aluminum at the CEI remains unclear. To address this, we utilized a combination of X-ray photoelectron and absorption spectroscopy to identify aluminum surface environments and extent of transition metal reduction for Ni-rich LiNi_0.8_Co_0.2−*y*_AlyO_2_ (0%, 5%, or 20% Al) layered oxide cathodes tested at 4.75 V under thermal stress (60 °C). For these tests, we compared the conventional LiPF_6_ salt with the more thermally stable LiBF_4_ salt. The CEI layers are inherently different between these two electrolyte salts, particularly for the highest level of Al-doping (20%) where a thicker (thinner) CEI layer is found for LiPF_6_ (LiBF_4_). Focusing on the aluminum environment, we reveal the type of surface aluminum species are dependent on the electrolyte salt, as Al-O-F- and Al-F-like species form when using LiPF_6_ and LiBF_4_, respectively. In both cases, we find cathode-electrolyte reactions drive the formation of a protective Al-F-like barrier at the CEI in Al-doped oxide cathodes.

## Introduction

Ni-rich layered oxides remain at the forefront of research on practical high-energy density Li-ion battery cathodes^1–4^. Several cathode degradation pathways hinder the full utilization of these systems, contributing to poor cycling performance beyond 4.3 V^5–8^ and limiting practical capacities of Ni-rich layered oxide cathodes to around 200 mAh/g^1,3^. At the cathode-electrolyte interface (CEI), cathode degradation involves a combination of oxygen loss^9–11^, phase transformations^12^, and transition metal dissolution^13,14^ at high voltages that promote capacity fade^6,7^ and impedance growth^15^.

Cathode and electrolyte modifications are often employed to inhibit these degradation pathways to improve high voltage stability^16–19^. A primary goal of these modifications is to overcome or limit problems that arise from the instability of the LiPF_6_ salt, which is known to decompose and form HF and POF_3_^20,21^ at high voltages or under thermal stress. These species are highly reactive, readily attack the layered oxide material, and play a key role in transition metal (TM) reduction and dissolution at the CEI^9,22^. Coating layers are used as a barrier to eliminate direct contact of these species with the TMs and thereby improve CEI stability. Out of the various materials tested as coating layers, aluminum oxide compounds have consistently demonstrated increased long-term stability and superior performance^17,18,23,24^. In contrast to the bare layered oxide surface, aluminum oxide coatings promote the formation of a thicker CEI layer consisting of P-O-F, Al-O-F, and TM-F species^23,25^. This thicker layer is considered to inhibit continued cathode degradation and subsequent dissolution. Al-doping is also known to improve high voltage performance^26,27^ leading to its use in both Co- and Ni-rich layered oxide cathodes^3,4^, though there remain few direct studies of aluminum at the CEI layer in these systems. X-ray photoelectron spectroscopy (XPS) measurements of the Al 2p/2s core regions provide a reliable probe of aluminum environments for coating layers^23,25,28^, yet for Ni-rich layered oxides the Ni 3p/3s core regions obscure the Al 2p/2s for Al-doped systems^29^.

In our previous work, we considered how the choice of electrolyte salt influences heat generation and cathode degradation for layered oxides under extreme conditions (4.75 V and 60 °C) when replacing the conventional LiPF_6_ salt with LiBF_4_^30^ which is more robust at higher voltages^31,32^ and elevated temperatures^33,34^. After 100 hrs of constant voltage (CV) holding at 4.75 V and 60 °C using the LiPF_6_ salt, the combined instability of the carbonaceous solvents and LiPF_6_ salt drove the onset of a complex exothermic reaction related to extreme TM dissolution for Co- and Ni-rich layered oxides^30^. Upon switching to LiBF_4_, we found less electrolyte decomposition species and TM reduction at the CEI and only a third of the TM dissolution observed when using LiPF_6_. Extended CV holding under these conditions still resulted in heat generation and impedance growth when using LiBF_4_ but no exothermic reaction was observed. In this case, drastic differences in CEI layer formation between these two salts were found for the LiNi_0.8_Co_0.15_Al_0.05_O_2_ (NCA) cathode^30^. Extension of this investigation to Al-doped and Al-free layered oxides can provide insight into the influence of Al-doping on the CEI layer formation and stability between these two electrolyte salts.

Here, we examine the CEI layer for layered oxides with 0% (LiNi_0.8_Co_0.2_O_2_/LNC), 5% (LiNi_0.8_Co_0.15_Al_0.05_O_2_/NCA), and 20% (LiNi_0.8_Al_0.2_O_2_/LNA) Al-doping held at 4.75 V (versus Li-metal) under thermal stress (60 °C) using either LiPF_6_ or LiBF_4_. In contrast to the minimal changes in the CEI layer composition for LNC (0% Al) and NCA (5% Al) under these conditions for the LiPF_6_ salt, we found a pronounced increase in the CEI layer thickness along with the formation of new surface species (Ni-F and P-O-F) for LNA (20% Al). Upon switching to LiBF_4_, minimal electrolyte decomposition species were observed to form under thermal stress, demonstrating the higher stability of LiBF_4_ in these conditions. Hard X-ray photoelectron spectroscopy measurements of the Al 1s were used to overcome the challenges associated with the Al 2s/2p core regions to provide qualitative and quantitative insight into aluminum environments. From these measurements, we observed the formation of new Al-O-F-like species at the CEI under thermal stress. The type of Al-O-F-like species were found to be distinct between the two salts with Al-O-F- and Al-F-like environments forming when using LiPF_6_ and LiBF_4_, respectively.

## Results

### CEI layer when using LiPF_6_

**Figure 1:**
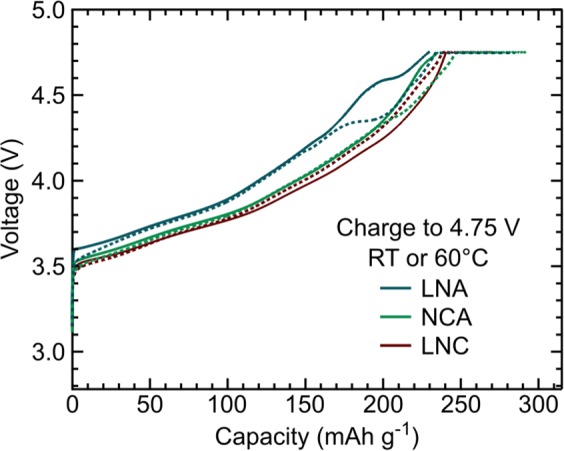
Electrochemical profiles for the LiNi_0.8_Co_0.2_O_2_ (LNC), LiNi_0.8_Co_0.15_Al_0.05_O_2_ (NCA), and LiNi_0.8_Al_0.2_O_2_ (LNA) compounds synthesized in-house when using LiPF_6_ as the electrolyte salt. Solid lines are used to represent electrodes charged to 4.75 V at 10 mAh/g and at RT with no CV hold. Dotted lines represent electrodes charged to 4.75 V at 25 mAh/g followed by a 10 hr CV hold at 4.75 V, all at 60 °C.

The influence of Al-doping on the CEI layer was evaluated for Ni-rich layered oxides (LiM_1−*y*_Al_*y*_O_2_ where M is TM = Ni/Co) by comparing electrodes under two testing conditions: (1) charged to 4.75 V at room temperature (RT) with no CV hold and (2) charged to 4.75 V at 60 °C with a 10 hr CV hold versus Li-metal. These electrochemical testing conditions will be referred to as RT and 60 °C, respectively. Electrochemical profiles for these two testing conditions for LNC, NCA, and LNA using LiPF_6_ are shown in Fig. 1. X-ray absorption spectroscopy (XAS) of the TM L_3_-edges and depth-dependent X-ray photoelectron spectroscopy (XPS) measurements were used to probe changes in TM oxidation states and chemical species at the CEI. HAXPES measurements of the Al 1s were performed to identify aluminum chemical environments. Peak fitting results for the Al 1s for the electrodes used within this study are given in Table S1. As initial Li-surface species, e.g. Li_2_CO_3_, LiOH, etc., are known to influence the surface reactivity of Ni-rich cathodes^30,35^, all systems were stored and processed in a dry-room post-synthesis to minimize the formation of these species^36^.

**Figure 2:**
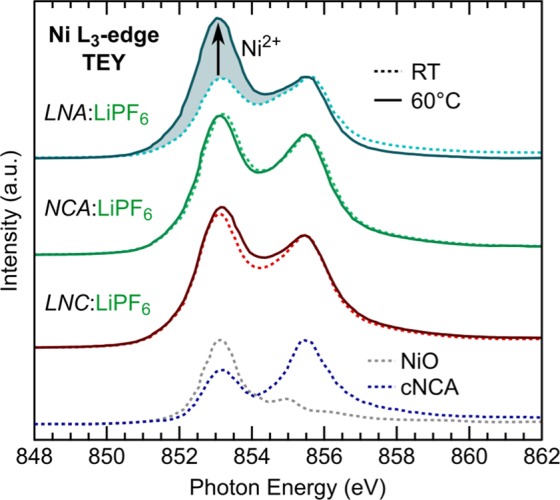
Ni L_3_-edge for LNA, NCA, and LNC electrodes charged to 4.75 V at RT with no CV and to 4.75 V at 60 °C and held at 4.75 V for 10 hrs at 60 °C. NiO (Ni^2+^) and *cNCA* electrode charged to 4.75 V at RT (~Ni^4+^ are included as reference spectra.

XAS measurements at the Ni L_3_-edge (Fig. 2) in surface sensitive total electron yield (TEY) mode were used to evaluate the extent of nickel surface reduction for the LNC (0% Al), NCA (5% Al), and LNA (20% Al) electrodes. Based on the charge capacity to 4.75 V at RT (Fig. 1), ~0.7 Li or more has been removed from these systems associated primarily with the Ni^3+/4+^ redox couple so that a ~Ni^4+^ oxidation state is expected. The theoretically calculated lineshape of Ni^4+^ shows a strong peak at 855.5 eV which can be contrasted with Ni^2+^ and Ni^3+^ lineshapes that have stronger peaks at lower energies^37^. Ni L_3_-edge spectra of NiO and a commercial NCA (*c*NCA) electrode charged to 4.75 V at RT are included in Fig. 2 as a Ni^2+^ and approximate Ni^4+^ reference, respectively. Each of the RT electrodes shows a stronger 853 eV peak compared to the *c*NCA reference spectra relative to higher energy features, which is associated with increased Ni^2+^. More Ni-reduction for the LNA, NCA, and LNC electrodes may be related to the smaller primary particles and higher surface area of these compounds relative to *c*NCA. For the 60 °C electrodes, only the LNA system shows a pronounced change in the Ni L_3_-edge lineshape associated with increased Ni-reduction compared to the RT electrodes (shading in Fig. 2). There is similarly limited change in the cobalt oxidation state for the LNC and NCA systems between 60 °C and RT electrodes from the Co L_3_-edge given in Fig. S1.

In Table 1, we give previously reported TM-dissolution percentages for 60 °C LNC, NCA, LNA, and *c*NCA electrodes that were determined from inductively coupled plasma-optical emission spectroscopy (ICP-OES)^38^. For the *c*NCA, there is less nickel and cobalt dissolution than the other compounds due to the lower surface area of this compound. Although only LNA electrodes show a pronounced increase in the extent of Ni-reduction from the Ni L_3_-edge between RT and 60 °C electrodes, a similar amount of nickel dissolution was observed for all three systems. The amount of Ni-dissolution in the Co-containing compounds (NCA and LNC) is actually higher than the parent LiNiO_2_ with 0.97% Ni-dissolution which was attributed to cobalt catalysizing the dissolution process^38^. Also, it is important to note that the disordered LNA material used for these measurements had much higher nickel dissolution then an ordered LNA sample with 20% Al-doping (0.28%)^38^. As discussed in this previous study^38^, this higher dissolution is likely a direct result of the disordering and not the higher Al-doping level.

**Table 1:** Ni and Co metal dissolution measurements by ICP-OES based on a Li-metal negative electrode and seperator retrieved from LiNi_0.8_Co_0.2−*y*_Al_*y*_O_2_ cells that were charged to 4.75 V at 25 mA/g and held at 4.75 V for 10 hrs at 60 °C.

Material	Average	Average
	%Ni dissolved	%Co dissolved
LNA (20% Al)	2.08	—
NCA (5% Al)	1.78	1.77
LNC (0% Al)	1.75	1.86
cNCA (5% Al)	0.82	0.68

These results were previously published in ref. ^38^.

**Figure 3:**
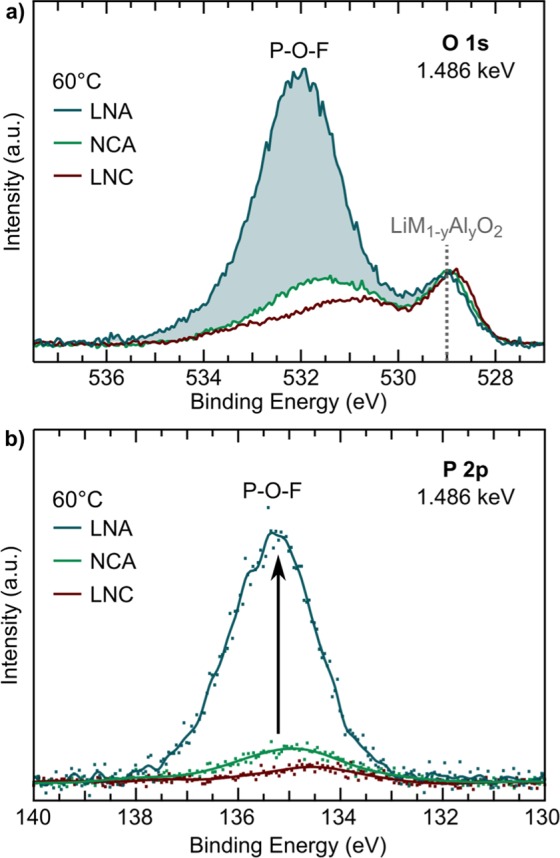
XPS measurements of the (**a**) O 1s and (**b**) P 2p core regions for LNA, NCA, and LNC electrodes charged to 4.75 V and held at 4.75 V for 10 hrs using the LiPF_6_ salt, all at 60 °C.

XPS measurements of the O 1s and P 2p core regions further reveal distinct differences between the 20% Al-doped LNA and the NCA/LNC 60 °C electrodes. In the O 1 s core region (Fig. 3a), the lower binding energy peak at 529 eV is assigned to lattice oxygen (LiM_1−*y*_Al_*y*_O_2_) and higher energy peaks, >530 eV, are assigned primarily to oxygen species in the near-surface region^39^. In the P 2p core region (Fig. 3b), the main peak at 135.2 eV is assigned to P-O-F species resulting from LiPF_6_ decomposition^23,25,28^. For the LNA electrode, we find an increase in the 532 eV O 1s peak and P-O-F P 2p peak compared to the NCA and LNC systems. Additional XPS measurements of the C 1s, Li 1s and F 1s core regions (Fig. S2) show minimal evidence of new lithium or carbon species forming between the 60 °C electrodes. Therefore, the 532 eV O 1s peak for LNA is primarily associated with the formation of surface P-O-F species based on its concurrent increase with the P 2p peak and lack of new carbon species. These same species form on the NCA surface after longer holding at 4.75 V^39^ but not as prominently on the LNC surface (Fig. S3), pointing towards the role of aluminum in driving these species formation at the surface. Competing effects between cobalt catalyzing dissolution and aluminum driving buildup of P-O-F species likely dicate the CEI composition of NCA.

**Figure 4:**
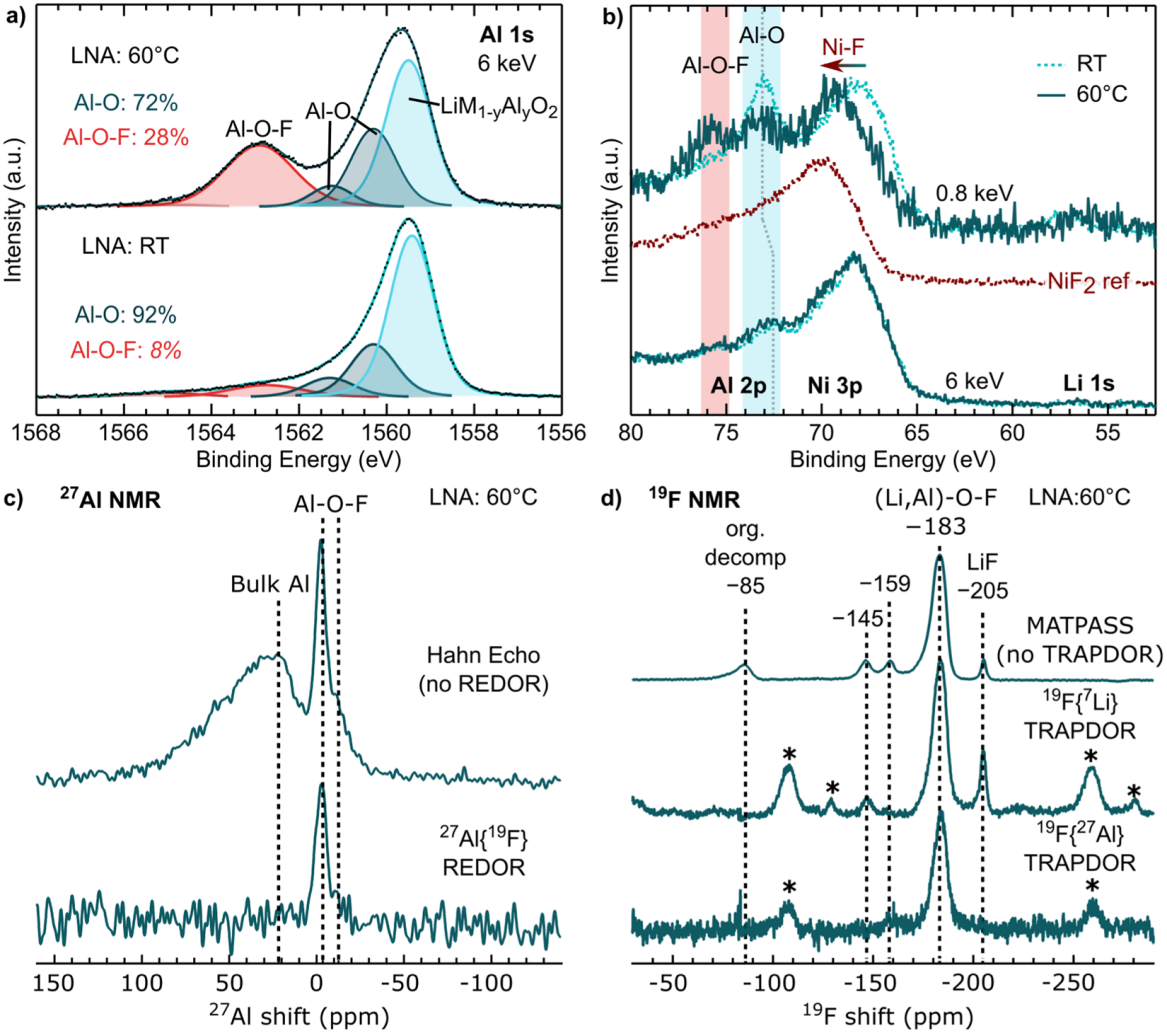
Measurements of RT and 60 °C LNA electrodes using the LiPF_6_ salt. (**a**) XPS measurements at 6 keV of the Al 1s core region along with corresponding peak fits. (**b**) XPS measurements at 0.8 and 6 keV of the Al 2p, Ni 3p and Li 1s regions along with a NiF_2_ reference. (**c**) ^27^Al NMR spectra, recorded at 16.4 T and 54 kHz MAS, using a Hahn echo pulse sequence (top) and the difference spectrum acquired with the ^27^Al {^19^F } REDOR method (bottom), showing ^27^Al resonances that arise from sites in proximity to ^19^F. (**d**) ^19^F NMR spectra recorded at 16.4 T and 50 kHz MAS, using MATPASS pulse sequence to suppress sidebands (top, isotropic slice shown), and difference spectra acquired using ^19^F {^7^Li } and ^19^F {^27^Al } TRAPDOR (middle and bottom, respectively), showing ^19^F resonances arising from sites spatially close to ^7^Li and ^27^Al, respectively. Asterisks denote spinning sidebands.

In our previous study, we highlighted how electrochemical and thermal stress during extended CV holding at high voltages and RT or 60 °C influenced surface aluminum environments for the *c*NCA system^29^. Here, we use a combination of photon-dependent XPS of the Al 1s and Al 2p core regions combined with specialized NMR methods to examine this further for Al-doped layered oxides. In Fig. 4a, we show peak fits of the Al 1s core region for RT and 60 °C LNA electrodes. The first three peaks at 1559.55, 1560.4, and 1561.2 eV are assigned to lattice LiM_1−*y*_Al_*y*_O_2_ and Al-O-like environments as indicated in the figure. The two higher binding energy Al-O-like peaks are primarily associated with surface environments based on our previous depth-dependent measurements of the Al 1s/Al 2p core regions^29^ and results shown within this current study. This may be an indication of aluminum segregation into aluminum oxide and lithium aluminate environments in the first few nm. Comparison of the *c*NCA, NCA, and LNA electrodes indicate that these Al-O species are material dependent with *c*NCA containing more of these environments Fig. S4 & Table S1)

For the LNA electrode held under thermal stress at 60 °C, we found the emergence of a higher binding energy peak at 1562.7 eV that is either absent or weak in RT LNA electrodes (Figs. 4a & S4). To help with the identification of this new higher binding energy peak, we performed depth-dependent XPS measurements of the Al 2p core region at 0.8 and 6 keV with probing depths of ~2.5 and ~23 nm, respectively. In sub-surface sensitive measurements (6 keV), we find the Al 2p unchanged between RT and 60 °C LNA electrodes. At the surface (0.8 keV), the 60 °C electrode shows a new Al 2p peak at 75.5 eV, which is between Al-O, LiAlO_2_ (73.6 eV) and Al_2_O_3_ (73.8 eV^25^), and AlF_3_ (76.6 eV). Therefore, these higher binding energy Al 1s (1562.6 eV) and Al 2p (75.5 eV) peaks are likely associated with various surface Al-O-F environments, similar to what has been found to form in previous aluminum oxide coating studies^23,28^. New peaks at 1562.7 eV are also observed for 60 °C NCA and *c*NCA electrodes when using LiPF_6_ (Fig. S4 & Table S1).

In order to provide additional insights into the nature of Al-O-F species observed in XPS, solid-state magic-angle spinning (MAS) ^27^Al and ^19^F NMR experiments were performed on a duplicate 60 °C LNA electrode. As we noted in our earlier NMR studies on *c*NCA^39^, the paramagnetic behavior of the electrode samples (due to sources of unpaired electron spin such as Ni^2+^) induces shortened spin-lattice (*T*_1_) and spin-spin (*T*_2_) relaxation constants, which can render spectral acquisition challenging and necessitates fast MAS rates and short recycle delays. Nonetheless, a ^27^Al Hahn echo spectrum (Fig. 4c, top) could be acquired and reveals two resonances at ~25 ppm (broad) and −3 ppm (sharp); the lower frequency resonance also shows a shoulder at roughly −10 ppm. The broad feature at ~25 ppm is assigned to bulk octahedral Al in the lattice, in agreement with Gaudin *et al*.^40^ and our prior ^27^Al NMR studies of cycled LCA and LNA phases^38^. This broad resonance arises from a distribution of ^27^Al shifts with differing numbers of Al or Ni neighbors^41^.

Interestingly, the sharp ^27^Al feature with a lower-frequency shoulder is in the shift range for six-coordinate Al in Al_2_O_3_ phases (~0 ppm) as well as that in hydrated AlF_3_ phases (−15 to −20 ppm);^42–44^ shifts at lower frequency are associated with greater numbers of neighboring F. To confirm if these features arise from Al in proximity to F, we performed ^27^Al {^19^F} rotational echo double-resonance (REDOR) measurements. The REDOR difference spectrum (Fig. 4c, bottom) shows ^27^Al resonances that experience dipolar coupling to nearby ^19^F. Features at −3 ppm and −10 ppm are observed corroborating our assignment of these resonances to Al-O-F species at the electrode surface. The presence of multiple sites likely reflects a distribution of aluminum oxyfluoride or hydroxyfluoride environments such as Al(OH)_*x*_F_6−*x*_ (1 < *x* < 6), as previously suggested by Chupas *et al*.^44^.

Additional support for assignment of F-containing surface species is provided by ^19^F NMR measurements (Fig. 4d). The topmost ^19^F spectrum, which is the isotropic slice from a magic-angle turning and phase-adjusted sideband separation (MATPASS) experiment^45^, shows at least five distinct resonances; the feature at −85 ppm is readily assigned to organic decomposition species such as OPF _2_OR and OPF(OR)_2_ (where R = Me, Et) in agreement with Campion *et al*.^21^, and corroborates the formation of P-O-F species observed in XPS measurements (Fig. 3). Moreover, the resonance at −205 ppm is consistent with LiF^46^.

The remaining ^19^F resonances (at −145, −159 and −183 ppm) are more difficult to rationalize in the first instance. We previously observed ^19^F NMR features at −145 and −183 ppm in our study of CEI formation on *c*NCA using a LiPF_6_-based electrolyte^39^, but at that time were unable to assign these to specific phases. To aid in assignment in the present work, we carried out ^19^F {^27^Al } and ^19^F {^7^Li } transfer population in double resonance (TRAPDOR) experiments, which selectively reveal ^19^F resonances in proximity to ^27^Al and ^7^Li, respectively. The resonance at −145 ppm is only observed in the ^19^F{^7^Li} TRAPDOR difference spectrum (Fig. 4d, middle), showing that this feature arises from a lithiated fluoride phase that is distinct from LiF (−204 ppm). Recent work by Clément *et al*.^47^ also reported a similar ^19^F shift at −144 ppm in cycled Li _1.15_Ni_0.45_Ti_0.3_Mo_0.1_O _1.85_F_0.15_, which was reversible upon cycling and was ascribed to lithiated fluoride on or at the electrode surface. The exact composition of this phase remains unclear, but the resonance does not appear in the ^19^F {^27^Al } TRAPDOR difference spectrum (Fig. 4d, bottom), so we assert that it is unrelated to the Al-O-F species. On the other hand, the feature resonating at −183 ppm appears in both TRAPDOR difference spectra, suggesting proximity to both ^27^Al and ^7^Li. On this basis, we assign this resonance to the Al-O-F species observed with XPS, which must also contain Li in close proximity. The ^19^F shift of −183 ppm is also reminiscent of, but distinct from, that of anhydrous AlF_3_ (−172 ppm)^44^. The width of this feature and the appearance of a higher-frequency shoulder is also consistent with a distribution of different local ^19^F environments expected from a disordered CEI. Finally, we tentatively assign the resonance at −159 ppm to residual HF arising from LiPF_6_ decomposition, possibly in a hydrogen-bonded complex, given that in prior work of Shenderovich *et al*.^48^, FHF- was observed to resonate at −155 ppm.

Returning to the 0.8 and 6 keV XPS measurements and focusing on the nickel environment (Fig. 4b), the main Ni 3p peak shifts towards higher binding energy for the 60 °C LNA electrode in surface sensitive 0.8 keV measurements. This shift is consistent with the formation of Ni-F environments based on comparison to NiF_2_ reference spectra. Under these testing conditions, new Ni-F environments are only observed for the LNA system and not found in similar depth-dependent XPS measurements of NCA electrodes (Fig. S5). For the NCA and LNA systems (Fig. 4b, Fig. S5), the Li 1s core region shows minimal surface species at high voltages and no change under thermal stress associated with LiPF_6_ electrolyte decomposition, i.e. no buildup of LiF or other lithium compounds at high voltages.

**Figure 5:**
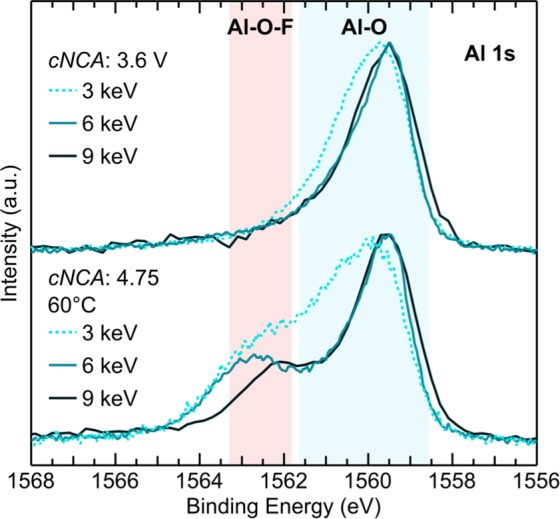
HAXPES measurements conducted at 3, 6, and 9 keV for commercial NCA electrodes (cNCA) charged to 3.6 V at RT, and to 4.75 V and held at 4.75 V for 10 hrs at 60 °C using the LiPF_6_ salt. The 9 keV measurements were collected using novel Lab-based Ga K *α* HAXPES system.

As part of our study on the formation of Al-O-F surface species (Fig. 5), we conducted depth-dependent measurements at 3, 6, and 9 keV on *c*NCA electrodes charged to 3.6 V at RT, and to 4.75 V at 60 °C with a 10 hr CV hold. The 3/6 keV and 9 (9.25) keV measurements were conducted at a synchrotron source (DLS: I09) and using the novel lab-based Ga K*α* X-ray source^49^, respectively. For the 3 keV measurements, a higher asymmetry of the main Al 1s peak is related to the presence of Al-O-like surface environments for the 3.6 V and 60 °C electrodes. Comparison of the 60 °C and 3.6 V electrodes reveals the emergence of a higher binding energy peak >1562 eV at all three photon energies related to the high voltage/temperature holding. The small variation in this higher energy peak is surprising given the wide range of probing depths from ~8 nm at 3 keV to ~31 nm at 9 keV and is evidence of a distribution of Al-O-F environments beyond the first 2–3 nm. In addition, this higher energy peak shifts lower in energy for the 9 keV measurements and suggests the type of Al-O-F environment changes from the surface to sub-surface. These measurements were used to demonstrate the high quality XPS spectra that can be acquired with the novel Ga K *α* X-ray lab source and, along with measurements of the Ni 2p and O 1s (Fig. S6), demonstrates the application of this system to the investigation of depth-dependent phenomena in battery materials.

### CEI layer when using LiBF_4_

**Figure 6:**
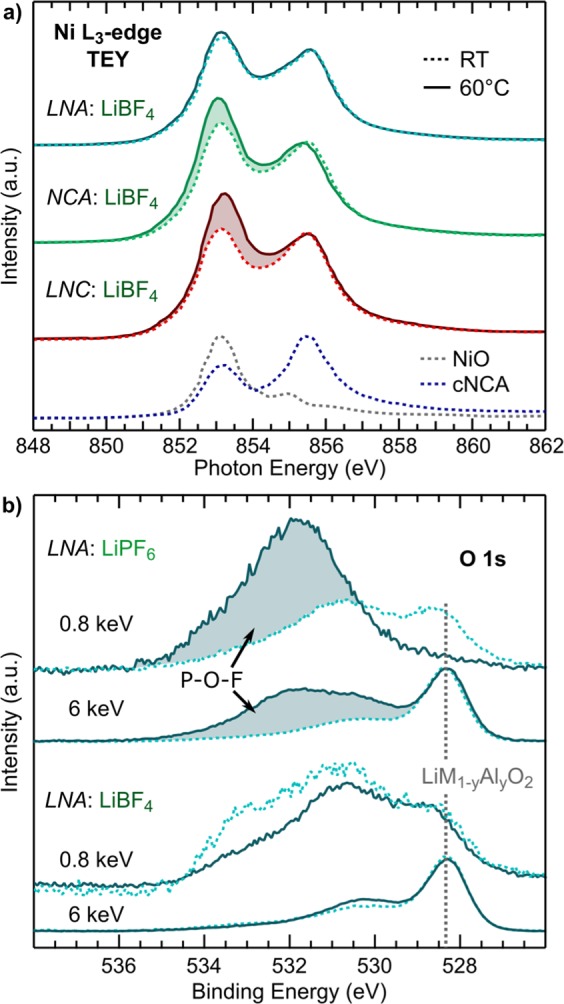
(**a**) Ni L_3_-edge measurements for LNA, NCA, and LNC RT and 60° electrodes using the LiBF_4_ salt. NiO (Ni^2+^) and cNCA charged to 4.75 V reference spectra are included. (**b**) Comparison of O 1s spectra measured at 0.8 and 6 keV for LNA electrodes charged to 4.75 V at RT and at 60 °C with a 10 hr CV hold using either the LiPF_6_ (top) or LiBF_4_ salt (bottom). The shading indicates the P-O-F formation at the CEI under thermal stress when using LiPF_6_.

For LNC, NCA, and LNA, similar electrochemical tests were conducted using the LiBF_4_ salt to examine how switching to LiBF_4_ impacts CEI formation. XAS measurements of the Ni L_3_-edge, shown in Fig. 6a, for RT and 60 °C electrodes were strikingly different from the LiPF_6_ case where LNA showed the most pronounced increase in surface reduction (Fig. 2). Compared with the RT electrodes, LNA and LNC 60 °C electrodes show the smallest and largest increase in the 853 eV peak associated with reduced Ni^2+^, respectively. An increase in Co-reduction was also observed for the NCA system when using the LiBF_4_ salt, which can be contrasted with the minimal changes observed when using LiPF_6_ (Fig. S1).

To further compare using LiPF_6_ and LiBF_4_, we performed XPS measurements at 0.8 and 6 keV of the O 1s core region, shown in Fig. 6b. For the LiPF_6_ case, there is a pronounced increase in surface oxygen peaks (shading in Fig. 6b) relative to the lattice LiM_1−*y*_Al_*y*_O_2_ peak for the 60 °C electrode compared to the RT electrode. The relative ratio of surface oxygen species to lattice oxygen increases by a factor of 12 and 2 for the 0.8 and 6 keV measurements, respectively. As identified in Fig. 3, this increase is related to the formation of P-O-F species when using LiPF_6_. Assuming this is a uniform overlayer, the ratio of the surface to lattice oxygen at 0.8 keV indicates a thickness of <2.25 nm. Yet, the change in the sub-surface 6 keV measurements cannot be accounted for by a 2.25 nm overlayer indicating it is not a uniform overlayer. Without using spatially resolved techniques it is difficult to approximate the distribution of this P-O-F layer at the surface. In contrast to the LiPF_6_ case, there is limited change in either the surface or sub-surface XPS measurements when using the LiBF_4_ salt. These trends in CEI layer composition when using LiPF_6_ and LiBF_4_ for the LNA system are further supported by complementary O K-edge measurements (Fig. S7). O K-edge spectra for LNC and NCA electrodes show only small changes for both electrolyte salts (Fig. S7).

**Figure 7:**
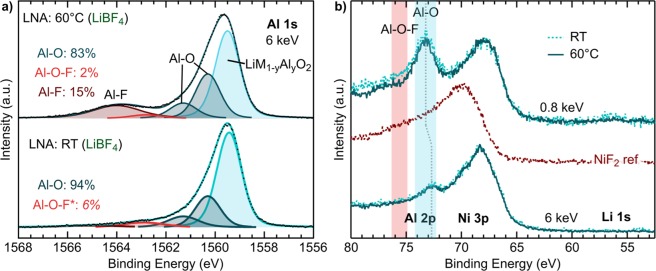
(**a**) HAXPES measurements at 6 keV of the Al 1s region and corresponding peak fits for LNA RT and 60 °C electrodes using the LiBF_4_ salt. (**b**) Depth-dependent XPS at 0.8 and 6 keV of the Al 2p, Ni 3p, and Li 1s regions of the LNA RT and 60 °C electrodes using the LiBF_4_ salt. The grey dotted line shows the shift in the Al 2p core level between surface (0.8 keV) and sub-surface (6 keV) measurements.

For the LNA system, we have found limited TM reduction or new electrolyte decomposition species at the CEI for the 60 °C electrode compared to the RT electrode when using LiBF_4_. We now turn to the Al 1s core region to determine how the LiBF_4_ salt influences aluminum-electrolyte reactions. Peak fits of the Al 1s core region for RT and 60 °C LNA electrodes using LiBF_4_ are shown in Fig. 7a. Surprisingly, a new higher binding energy peak is found at 1564.0 eV for the 60 °C electrode over 1 eV higher than the Al-O-F peak that forms when using LiPF_6_. Assuming a similar binding energy trend in the Al 1s core region as in the Al 2p for Al-O, Al-O-F, and Al-F environments, this higher binding energy peak is likely associated with environments that are more Al-F-like when using LiBF_4_. In the Al 2p region, no new peak is distinguishable for the 60 °C electrode above the Al-O peak at 0.8 and 6 keV (Fig. 7b). This further highlights the benefit of using the Al 1s as the new Al-F-like environment is not distinguishable in the Al 2p, even with 20% Al-doping.

**Figure 8:**
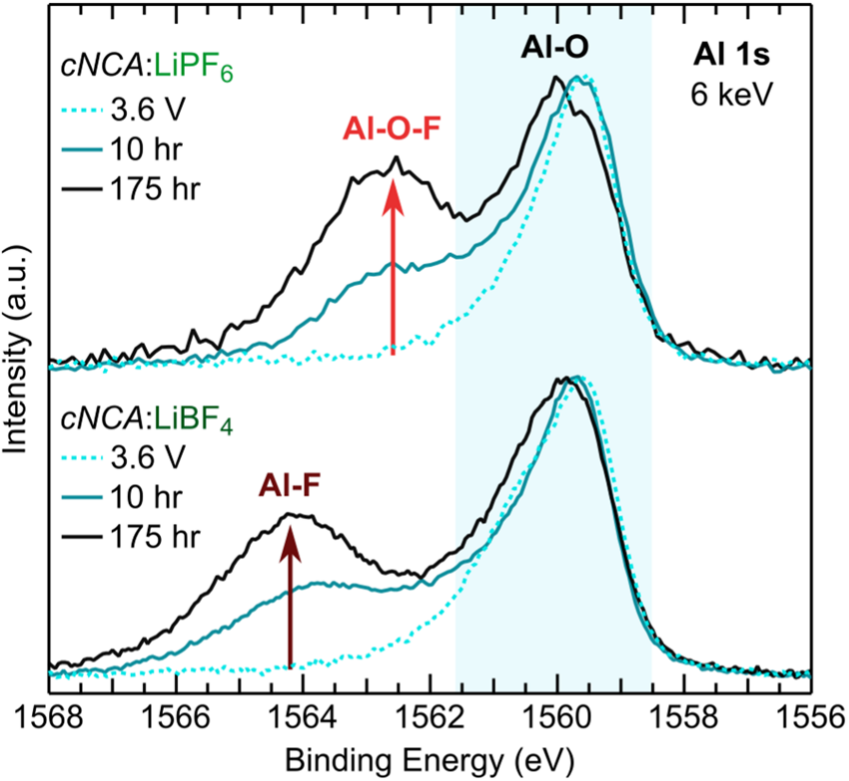
Al 1s core region for electrodes using cNCA charged to 3.6 V at RT, 4.75 V at 60 °C with a 10 hr or a 175 hr CV hold at 4.75 V and 60 °C. The arrows indicate the formation of a new aluminum surface environment with holding at high voltage and temperature that is dependent on the choice of electrolyte salt.

Additional high voltage CV tests for 10 and 175 hrs at 4.75 V and 60 °C for *c*NCA were conducted to identify whether this salt dependence is observed across Al-doped systems. As noted in the introduction, the half-cell undergoes a unique exothermic reaction after around 100 hrs when using LiPF_6_ but not when using LiBF_4_^30^. The electrodes held for 10 hrs were measured in the discharged state (2.7 V vs Li-metal) while the electrodes held for 175 hrs were not due to the effect of the exothermic reaction in the LiPF_6_ case on the open circuit voltage^30^. In Fig. 8, we show XPS measurements of the Al 1s core region at 6 keV for these *c*NCA electrodes using either LiBF_4_ or LiPF_6_. Reference electrodes charged to 3.6 V at RT for each electrolyte are included for comparison. For the 3.6 V electrodes, no clear higher binding Al 1s peaks (>1562 eV) were observed for either salt. For the LiPF_6_ case, a new peak at 1562.7 eV is observed for the 10 and 175 hr 60 °C electrodes. For the LiBF_4_ case, the new peak is over 1 eV higher at 1564.0 eV for both 60 °C electrodes and at a similar binding energy as the Al-F-like environment in the LNA case. This consistency for LNA and *c*NCA demonstrates a clear dependence of the aluminum-electrolyte reactions at the CEI on the choice of electrolyte salt.

## Discussion

From a comprehensive study of Ni-rich LiNi_0.8_Co_0.2−*y*_Al_*y*_O_2_ electrodes held under thermal stress at 4.75 V, we have identified a clear influence of Al-doping on the formation of the CEI layer at high voltages/temperatures. When using LiPF_6_, aluminum is found to drive the formation of a thicker CEI layer consisting primarily of P-O-F, Ni-F (reduced TMs), and Al-O-F surface species, similar to what has been observed upon cycling in aluminum oxide coating studies^23,25^. In these studies, the thicker CEI layer was considered to improve cycling by acting as a protective barrier against continued cathode degradation induced by the decomposition of the LiPF_6_ salt. It seems a similar mechanism occurs in Al-doped systems based on suppressed TM-dissolution with Al-doping^26,38,50^. In the LiBF_4_ case, there was minimal buildup of LiBF_4_-decomposition species under thermal stress for LNA, reflecting the higher chemical stability of the LiBF_4_ salt in these conditions compared with LiPF_6_^30^. The absence of highly reactive POF_3_ analogs (e.g. BOF) in the LiBF_4_ electrolyte in these conditions circumvents reactions that enduce the formation of a thick layer in the LiPF_6_ case. This pronounced difference between these two salts rationalizes the improved high voltage performance of the spinel systems when using LiBF_4_^31^.

Using depth-dependent XPS of the Al 1s/Al 2p and NMR measurements, we provided a more detailed look into aluminum chemical environments at the surface of layered oxide cathodes. This methodology, combining the surface and elemental sensitivity of XPS with the local chemical sensitivity of the NMR REDOR and TRAPDOR measurements, enabled the assignment of aluminum chemical environments and characterization of the formation of these environments. While the tendency of aluminum segregation in the bulk is likely at higher doping levels in NCA-type systems^41,51^, our results suggest there may be some aluminum segregation in the first few nm of uncycled materials based on the presence of Al-O peaks that are distinct from bulk LiM_1−*y*_Al_*y*_O_2_. More work is needed to understand the exact chemical assignment of these surface Al-O environments and their dependence on synthesis, storage, and electrochemical testing.

Comparing Al-O-F-like environments at the CEI for the two electrolyte salts, our results demonstrate the dependence of aluminum-electrolyte reactions on the selected electrolyte salt: LiPF_6_ (Al-O-F-like) and LiBF_4_ (Al-F-like). For coating studies, Al-O-F environments have primarily been considered to form through reactions with HF^52^. While trace HF is present in practical operating conditions, increased HF formation is likely under our electrochemical testing conditions given the combined effect of thermal stress and high voltage on electrolyte decomposition for LiPF_6_ salts^21^. Under these conditions, layered oxides can catalytically drive the breakdown of carbonaceous solvents leading to increased water generation in the electrolyte^53^. Water generated through these processes react with fluoride salts, particularly LiPF_6_, to form HF and POF_3_ in the electrolyte^33^. Aluminum oxide coatings are widley considered to act as HF scavengers^17,52,54^ with the following reaction pathway between an Al_2_O_3_ coating layer and HF proposed by Myung *et al*.:^52^

1 $$A{l}_{2}{O}_{3}+2HF\to A{l}_{2}{O}_{2}{F}_{2}+{H}_{2}O$$

2 $$A{l}_{2}{O}_{2}{F}_{2}+2HF\to A{l}_{2}O{F}_{4}+{H}_{2}O$$

3 $$A{l}_{2}O{F}_{4}+2HF\to 2Al{F}_{3}+{H}_{2}O$$

Looking in more detail at the possible reactions, ab initio calculations found HF reactions at the alumina surface with hydroxyl termination are highly exothermic and result in the formation of AlF(OH) termination without producing H_2_O as a byproduct^22^. This work supported the notion that aluminum oxide coatings play a beneficial role at the cathode surface as HF scavengers that inhibit cyclic cathode degradation pathways^22^. A similar HF scavenger role is likely in Al-doped systems given our evidence for Al-O-F formation, though additional aluminum-electrolyte interactions, such as with highly reactive POF_3_ in LiPF_6_ electrolyte, may need to be considered to understand the dependence of Al-O-F species formation on choice of electrolyte salt found in this study. Although we do not provide an exact assignment of these species, the combined insights from our XPS and NMR measurements can be used to inform further investigation and modeling of aluminum surface environments.

In both the LiPF_6_ and LiBF_4_ cases, the evolution of surface aluminum towards Al-F-like environments may give rise to a stable CEI barrier against continued cathode degradation pathways. This may be similar to the benefits of using an AlF_3_ surface coating layer, which is considered a beneficial modification for improving the electrochemical and thermal stability of oxide cathodes at high states of charge^55,56^. Further consideration of the formation of these types of surface aluminum environments and their impact on CEI stability and cathode performance for Al-doped and aluminum coated layered oxides is warranted given the widespread application of these modifications to Ni-rich (NCA and NMC) and Li-rich layered oxides.

## Conclusion

From our focus on RT and 60 °C LNC, NCA, and LNA electrodes, we established the influence of aluminum and electrolyte salt (LiPF_6_ vs. LiBF_4_) on the CEI layer formation under electrochemical and thermal stress. Surface aluminum was identified to contribute to the formation of a thicker CEI layer when using LiPF_6_ salt consisting of P-O-F and Ni-F species, as shown for the LNA system with 20% Al-doping. In the LiBF_4_ case, we found limited additional CEI layer species in all three systems due to the increased stability of the LiBF_4_ salt at higher voltages and temperatures. Combining NMR measurements with changes in the Al 1s core region as a sensitive probe of aluminum down to 5% Al-doping, we highlighted the evolution of aluminum at the CEI towards Al-O-F- and Al-F-like species when using LiPF_6_ and LiBF_4_, respectively. While aluminum drives the formation of a surface layer that may protect against continuous cathode degredation in both electrolytes, the reaction mechanism is strongly dependent on the choice of electrolyte. The sensitivity of the aluminum environment on the choice of electrolyte salt can help inform the rational design of coatings that can improve capacity retention at high voltages.

## Methods

### Material synthesis

LiNi_0.8_Co_0.2_O_2_ (LNC), LiNi_0.8_Co_0.15_Al_0.05_O_2_ (NCA), and LiNi_0.8_Al_0.2_O_2_ (LNA) compounds were synthesized in-house as previously described^38^. The LNA compound used was referred to as disordered (DO) in our previous study due to poor transition metal mixing and higher phase segregation, including a ~1.3–2.0% *γ*-LiAlO_2_ impurity phase^38^. These in-house synthesized compounds mostly do not agglomerate and each system had similarly sized primary particles around 200 nm in diameter. Commercially produced LiNi_0.8_Co_0.15_Al_0.05_O_2_ (*c*NCA) was also tested for comparison to build upon our previous studies of the CEI layer for this system^29,30,39^. The *c*NCA material has larger primary particles from 300 nm to 1 *μ*m in diameter as previously reported^57^. All samples were stored in a dry-room post-synthesis to limit the formation of Li_2_CO_3_ and other Li-species on the cathode surface^36^.

### Electrode preparation and electrochemical characterization

Following the same methodology described in refs. ^29,30,39^, dried LiNi_0.8_Co_0.2−*y*_Al_*y*_O_2_ powder was mixed in an argon-filled glovebox with 2.5 wt% carbon black (SuperP, MMM) using a mortar and pestle. Powder cells (2032 Hohsen) were assembled using lithium metal (FMC) negative electrode with a combination of glass fiber (Whatman), polyolefin (Celgard) and PVDF-HFP depending on the test temperature, with 1 M LiPF_6_ or 1 M LiBF_4_ in ethylene carbonate:dimethyl carbonate (EC:DMC) (1:1 volume ratio) electrolyte (BASF).

Two primary testing conditions were used for these electrodes throughout the study: (1) charged to 4.75 V at 10 mAh/g and at 24 °C (room temperature/RT) with no constant voltage (CV), and (2) charged to 4.75 V at 25 mAh/g at 60 °C with a 10 hr CV hold at 4.75 V and at 60 °C. Electrodes charged to 3.6 V at 24 °C (RT) were used as a reference for each of the Al-doped systems. Electrochemical profiles for these two testing conditions for the LNC, NCA, and LNA electrodes using LiPF_6_ are shown in Fig. 1.

### X-ray spectroscopy

For all X-ray spectroscopy measurements, disassembled powder electrodes were mounted on conductive tape and transferred using vacuum suitcase set-ups between the glovebox and vacuum chamber or using a glove-bag setup in case of HAXPES prototype system to avoid air exposure. Powder electrodes were not washed prior to measurements to retain CEI species. Lab based X-ray photoelectron spectroscopy (XPS) measurements were performed using the Al K *α* and novel Ga K *α* sources^49^ with monochromated energies of 1.486 keV and 9.25 keV (referred to as 9 keV in text). Depth-dependent XPS measurements were conducted at beamline I09 at the Diamond Light Source Ltd. (DLS), UK using photon energies of 0.8, 3, and 6 keV. Hard X-ray photoelectron spectroscopy (HAXPES) measurements at 9 keV were conducted using the novel laboratory-based HAXPES prototype system with Ga K *α* monochromated source of 9.25 keV^49^. For each core region, the inelastic mean free path (IMFP) was calculated using the TPP-2M method^58^ using the NIST electron IMFP database^59^. Probing depths are approximately 3x the IMFP (from which 95% of the signal originates). The C 1 s peak for carbon black was aligned to 284.5 eV for energy calibration. Reference XPS measurements were performed for the NiF_2_, AlF_3_, and *γ*-LiAlO_2_ systems.

Soft X-ray absorption spectroscopy (XAS) measurements were performed in total electron yield (TEY) mode at beamline I09 in tandem with the depth-dependent XPS measurements. For the O K-edge spectra, photon energies were normalized to a TiO_2_ reference. For the Co and Ni L-edges, photon energies were normalized to a Ni-metal reference. The Co L_3_-edge and Ni L_3_-edge spectra were scaled to the peaks at ~780 eV and ~855 eV, respectively.

### Nuclear magnetic resonance (NMR) characterization

Solid-state ^19^F, ^27^Al and ^7^Li magic-angle spinning (MAS) NMR measurements were carried out on a 16.4 T Bruker Avance III 700 MHz spectrometer (University of Cambridge), operating at a Larmor frequency of 658.63 MHz (for ^19^F), 182.41 MHz (for ^27^Al), or 272.07 MHz (for ^7^Li), using a double-resonance Bruker 1.3 mm HX probe. The binder-free powder samples were packed into airtight 1.3 mm ZrO_2_ rotors within an Ar-filled glovebox, thus avoiding exposure to ambient atmosphere. Spectra were acquired under a MAS frequency of 50 kHz (for ^19^F experiments) or 54 kHz (for ^27^Al experiments). Typical one-dimensional spectra were acquired with a rotor-synchronized Hahn echo pulse sequence of the form (*π*/2)_*x*_-*τ*-(*π*)_*y*_-*τ*-acquire. The *π*/2 pulse length was 1.67 *μ*s (for ^19^F) or 0.75 *μ*s (for ^27^Al), and the recycle delay was 250 ms (for ^19^F) or 10 ms (for ^27^Al). When necessary to suppress sidebands (typically for the ^19^F measurements with broad spinning sideband manifolds), projection magic-angle turning and phase-adjusted sideband separation (pj-MATPASS) NMR spectra were recorded^45^. The ^19^F and ^27^Al chemical shifts were externally referenced to AlF_3_ (−172 ppm for ^19^F and −15.5 ppm for ^27^Al);^44^ the ^7^Li chemical shifts were externally referenced to LiF at −1 ppm^60^. NMR data were processed using Bruker TopSpin 3.2.

### ^27^Al{^19^F} REDOR NMR

The ^27^Al{^19^F} rotational echo double resonance (REDOR) experiments^61^ were performed by measuring ^27^Al spin-echo spectra with and without on-resonance irradiation of the ^19^F nuclei by a series of *π* pulses (3.34 *μ*s) at half-integral multiples of the rotor period; a total of 3 rotor periods were used, where the MAS rate was 54 kHz. Difference spectra were obtained by post-processing in TopSpin 3.2 by subtracting the spectra acquired with and without ^19^F irradiation.

### ^19^F{^27^Al} and ^19^F{^7^Li} TRAPDOR NMR

The ^19^F{^27^Al} and ^19^F{^7^Li} transfer population in double resonance (TRAPDOR) experiments^62^ were performed by acquiring ^19^F spin-echo spectra with and without continuous, on-resonance irradiation of the ^27^Al (or ^7^Li) nuclei throughout the evolution time which was equal to 1 rotor period; the MAS rate was 50 kHz. Difference spectra were obtained in an analogous way to the REDOR NMR experiments.

## Supplementary information

Supplementary Information

## References

[1] Choi JW, Aurbach D (2016). Promise and reality of post-lithium-ion batteries with high energy densities. Nat. Rev. Mater..

[2] Xu J, Lin F, Doeff M, Tong W (2016). A review of Ni-based layered oxides for rechargeable Li-ion batteries. J. Mater. Chem. A.

[3] Myung ST (2017). Nickel-rich layered cathode materials for automotive lithium-ion batteries: achievements and perspectives. ACS Energy Lett..

[4] Radin MD (2017). Narrowing the gap between theoretical and practical capacities in Li-ion layered oxide cathode materials. Adv. Energy Mater..

[5] Lin F (2014). Surface reconstruction and chemical evolution of stoichiometric layered cathode materials for lithium-ion batteries. Nat. communications.

[6] Hwang S, Kim DH, Chung KY, Chang W (2014). Understanding local degradation of cycled Ni-rich cathode materials at high operating temperature for Li-ion batteries. Appl. Phys. Lett..

[7] Lin F (2016). Metal segregation in hierarchically structured cathode materials for high-energy lithium batteries. Nat. Energy.

[8] Liu H (2017). Intergranular cracking as a major cause of long-term capacity fading of layered cathodes. Nano Lett..

[9] Gauthier M (2015). The electrode-electrolyte interface in Li-ion batteries: current understanding and new insights. The J. Phys. Chem. Lett..

[10] Jung R, Metzger M, Maglia F, Stinner C, Gasteiger HA (2017). Oxygen release and its effect on the cycling stability of LiNi_*x*_Mn_*y*_Co_*z*_O_2_ (NMC) cathode materials for Li-ion batteries. J. The Electrochem. Soc..

[11] Mu L (2018). Oxygen release induced chemomechanical breakdown of layered cathode materials. Nano Lett..

[12] Hwang S (2014). Investigation of changes in the surface structure of Li*x*Ni0.8Co0.15Al0.05O2 cathode materials induced by the initial charge. Chem. Mater..

[13] Amatucci G, Tarascon JM, Klein LC (1996). Cobalt dissolution in LiCoO_2_-based non-aqueous rechargeable batteries. Solid State Ionics.

[14] Zheng H, Sun Q, Liu G, Song X, Battaglia VS (2012). Correlation between dissolution behavior and electrochemical cycling performance for LiNi_1=3_Co_1=3_Mn_1=3_O_2_-based cells. J. Power Sources.

[15] Sallis S (2016). Surface degradation of Li_1−*x*_Ni_0.80_Co_0.15_Al_0.05_O_2_ cathodes: Correlating charge transfer impedance with surface phase transformations. Appl. Phys. Lett..

[16] Sun YK (2009). High-energy cathode material for long-life and safe lithium batteries. Nat. Mater..

[17] Chen Z, Qin Y, Amine K (2010). Role of surface coating on cathode materials for lithium-ion batteries. J. Mater. Chem..

[18] Scott ID (2011). Ultrathin coatings on nano-LiCoO_2_ for Li-ion vehicular applications. Nano Lett..

[19] Xia J (2016). A study of Li-ion cells operated to 4.5 V and at 55 °C. J. The Electrochem. Soc..

[20] Xu K (2004). Nonaqueous liquid electrolytes for lithium-based rechargeable batteries. Chem. Rev..

[21] Campion, C. L., Li, W. & Lucht, B. L. Thermal decomposition of LiPF_6_-based electrolytes for lithium-ion batteries. *J. The Electrochem. Soc*. **152**, A2327 Note that in the work of Campion et al., a different ^19^F chemical shift scale is used; to convert to the convention used here, 140 ppm must be added to the shifts in the prior work (2005).

[22] Tebbe JL, Holder AM, Musgrave CB (2015). Mechanisms of LiCoO_2_ Cathode Degradation by Reaction with HF and Protection by Thin Oxide Coatings. ACS Appl. Mater. Interfaces.

[23] Lu YC, Mansour AN, Yabuuchi N, Shao-Horn Y (2009). Probing the origin of enhanced stability of AlPO_4_ nanoparticle coated LiCoO_2_ during cycling to high voltages: Combined XRD and XPS studies. Chem. Mater..

[24] Yano A (2015). Surface structure and high-voltage charge/discharge characteristics of Al-Oxide coated LiNi_1=3_Co_1=3_Mn_1=3_O_2_ cathodes. J. The Electrochem. Soc..

[25] Verdier S (2007). XPS study on Al_2_O_3_- and AlPO_4_-coated LiCoO_2_ cathode material for high-capacity Li ion batteries. J. The Electrochem. Soc..

[26] Myung ST, Kumagai N, Komaba S, Chung HT (2001). Effects of Al doping on the microstructure of LiCoO_2_ cathode materials. Solid State Ionics.

[27] Chen CH (2004). Aluminum-doped lithium nickel cobalt oxide electrodes for high-power lithium-ion batteries. J. Power Sources.

[28] Baggetto L, Dudney NJ, Veith GM (2013). Surface chemistry of metal oxide coated lithium manganese nickel oxide thin film cathodes studied by XPS. Electrochimica Acta.

[29] Lebens-Higgins ZW (2017). Electrochemical and thermal stress of LiNi_0.8_Co_0.15_Al_0.05_O_2_ electrodes: evolution of aluminum surface environments. ECS Transactions.

[30] Faenza NV (2017). Electrolyte-induced surface transformation and transition-metal dissolution of fully delithiated LiNi_0.8_Co_0.15_Al_0.05_O_2_. Langmuir.

[31] Pereira N, Ruotolo MC, Lu MY, Badway F, Amatucci GG (2016). Elevated temperature performance of high voltage Li_1+*y*_Mn_1.5_Ni_0.5_O_4−*x*_F_*x*_ spinel in window-shifted Li-ion cells. J. Power Sources.

[32] Doi T (2018). Fluoroalkyl ether-diluted dimethyl carbonate-based electrolyte solutions for high-voltage operation of LiNi_0.5_Co_0.2_Mn_0.3_O_2_ electrodes in lithium ion batteries. Sustain. Energy & Fuels.

[33] Du Pasquier A (1999). An update on the high temperature ageing mechanism in LiMn_2_O_4_-based Li-ion cells. J. Power Sources.

[34] Sonoda T, Okada S, Gopukumar S, Yamaki J-I, Hong E-S (2004). Thermal stability of electrolytes with mixtures of LiPF_6_ and LiBF4 used in lithium-ion cells. J. The Electrochem. Soc..

[35] Cho D-H (2014). Effect of residual lithium compounds on layer Ni-rich Li[Ni_0.7_Mn_0.3_]O_2_. J. Electrochem. Soc..

[36] Faenza NV (2017). Growth of ambient induced surface impurity species on layered positive electrode materials and impact on electrochemical performance. J. The Electrochem. Soc..

[37] Qiao R (2017). Transition-metal redox evolution in LiNi_0.5_Mn_0.3_Co_0.2_O_2_ electrodes at high potentials. J. Power Sources.

[38] Faenza NV (2018). Phase evolution and degradation modes of R3m Li_*x*_Ni_1−*y*−*z*_Co_*y*_Al_*z*_O_2_ electrodes cycled near complete delithiation. Chem. Mater..

[39] Lebens-Higgins ZW (2018). Evolution of the electrode-electrolyte interface of LiNi_0.8_Co_0.15_Al_0.05_O_2_ electrodes due to electrochemical and thermal stress. Chem. Mater..

[40] Gaudin E (2001). Cobalt (III) effect on ^27^Al NMR chemical shifts in LiAl_*x*_Co_1−*x*_O_2_. The J. Phys. Chem. B.

[41] Trease NM (2016). Identifying the distribution of Al 3+ in LiNi_0.8_Co_0.15_Al_0.05_O_2_. Chem. Mater..

[42] Rosina KJ (2012). Structure of aluminum fluoride coated Li[Li_1=9_Ni_1=3_Mn_5=9_]O_2_ cathodes for secondary lithium-ion batteries. J. Mater. Chem..

[43] König R (2010). Spectroscopic characterization of crystalline AlF3 phases. J. Fluor. Chem..

[44] Chupas PJ, Corbin DR, Rao VNM, Hanson JC, Grey CP (2003). A combined solid-state NMR and diffraction study of the structures and acidity of fluorinated aluminas: implications for catalysis. The J. Phys. Chem. B.

[45] Hung I, Zhou L, Pourpoint F, Grey CP, Gan Z (2012). Isotropic high field NMR spectra of Li-ion battery materials with anisotropy >1 MHz. J. Am. Chem. Soc..

[46] Michan AL (2016). Solid electrolyte interphase growth and capacity loss in silicon electrodes. J. Am. Chem. Soc..

[47] Clément RJ, Kitchaev D, Lee J, Ceder G (2018). Short-range order and unusual modes of nickel redox in a fluorinesubstituted disordered rocksalt oxide lithium-ion cathode. Chem. Mater..

[48] Shenderovich IG (2003). Low-temperature NMR studies of the structure and dynamics of a novel series of acid-base complexes of HF with collidine exhibiting scalar couplings across hydrogen bonds. J. Am. Chem. Soc..

[49] Regoutz, A. *et al*. A novel laboratory-based hard X-ray photoelectron spectroscopy system. *Rev. Sci. Instruments* **89** (2018). 10.1063/1.5039829 30068129

[50] Li J, Manthiram A (2019). A comprehensive analysis of the interphasial and structural evolution over long-term cycling of ultrahigh-nickel cathodes in lithium-ion batteries. Adv. Energy Mater..

[51] Dogan, F., Vaughey, J. T., Iddir, H. & Key, B. Direct observation of lattice aluminum environments in Li ion cathodes LiNi_1−*y*−*z*_Co_*y*_Al_*z*_O_2_ and Al-doped LiNi_*x*_Mn_*y*_Co_*z*_O_2_ via ^27^Al MAS NMR spectroscopy. *ACS Appl. Mater. & Interfaces* **8**, 167–9=16717 (2016). 10.1021/acsami.6b04516 27299505

[52] Myung S-T, Izumi K, Komaba S, Sun Y-K (2005). Role of alumina coating on Li-Ni-Co-Mn-O particles as positive electrode material for lithium-ion batteries. Chem. Mater..

[53] Kumai K, Miyashiro H, Kobayashi Y, Takei K, Ishikawa R (1999). Gas generation mechanism due to electrolyte decomposition in commercial lithium-ion cell. J. Power Sources.

[54] Jung Y-SS (2010). Enhanced stability of LiCoO_2_ cathodes in lithium-ion batteries using surface modification by atomic layer deposition. J. The Electrochem. Soc..

[55] Lee SH, Yoon CS, Amine K, Sun YK (2013). Improvement of long-term cycling performance of Li[Ni_0.8_Co_0.15_Al_0.05_]O_2_ by AlF_3_ coating. J. Power Sources.

[56] Hu E (2018). Evolution of redox couples in Li- and Mn-rich cathode materials and mitigation of voltage fade by reducing oxygen release. Nat. Energy.

[57] Liu H (2018). Identifying the chemical and structural irreversibility in LiNi_0.8_Co_0.15_Al_0.05_O_2_ – a model compound for classical layered intercalation. J. Mater. Chem. A.

[58] Shinotsuka H, Tanuma S, Powell CJ, Penn DR (2015). Calculations of electron inelastic mean free paths. X. Data for 41 elemental solids over the 50 eV to 200 keV range with the relativistic full Penn algorithm. Surf. Interface Analysis.

[59] Powell, C. J. & Jablonski, A. NIST Electron Inelastic-Mean-Free-Path Database. Version 1.2, SRD 71 (National Institute of Standards and Technology, Gaithersburg, MD, 2010).

[60] Michan AL (2016). Fluoroethylene carbonate and vinylene carbonate reduction: Understanding lithium-ion battery electrolyte additives and solid electrolyte interphase formation. Chem. Mater..

[61] Gullion, T. *Modern Magnetic Resonance* (Springer, Netherlands: Dordrecht, 2006).

[62] Grey CP, Vega AJ (1995). Determination of the quadrupole coupling constant of the invisible aluminum spins in zeolite HY with ^1^H/^27^Al TRAPDOR NMR. J. Am. Chem. Soc..

