# Supplementary material for: Surface Chemistry Dependence on Aluminum Doping in Ni-rich LiNi0.8Co0.2−yAlyO2 Cathodes
Source: Sci Rep. 2019 Nov 27;9:17720. doi: 10.1038/s41598-019-53932-6 (PMC6881288; doi:10.1038/s41598-019-53932-6)
Supplement: Supplementary file 1 — Supplementary Information [file 41598_2019_53932_MOESM1_ESM.pdf]

Supporting Information: Surface Chemistry Dependence on Aluminum Doping in Ni-rich $LiNi_{0.8}Co_{0.2-y}Al_yO_2$ Cathodes

Zachary W. Lebens-Higgins,[†] David M. Halat,[‡,¶] Nicholas V. Faenza,[§] Matthew J Wahila,[†] Manfred Mascheck,[‖] Tomas Wiell,[‖] Susanna K. Eriksson,[‖] Paul Palmgren,[‖] Jose Rodriguez,[⊥] Fadwa Badway,[§] Nathalie Pereira,[§] Glenn G. Amatucci,[§] Tien-Lin Lee,[#] Clare Grey,[‡] and Louis F. J. Piper[*,⊥,@]

†*Department of Physics, Applied Physics and Astronomy, Binghamton University, Binghamton, NY 13902, USA*

‡*Department of Chemistry, University of Cambridge, Lensfield Road, Cambridge, CB2 1EW, U.K.*

¶*Current Address: Department of Chemical and Biomolecular Engineering, University of California, Berkeley, CA 94720, United States*

§*Energy Storage Research Group, Department of Materials Science and Engineering, Rutgers University, North Brunswick, NJ 08902, United States*

‖*Scienta Omicron AB, P.O. Box 15120, 750 15 Uppsala, Sweden*

⊥*Department of Physics, Applied Physics and Astronomy, Binghamton University, Binghamton, New York 13902, USA*

#*Diamond Light Source Ltd., Diamond House, Harwell Science and Innovation Campus, Didcot, Oxfordshire OX11 0DE, UK*

@*Materials Science & Engineering, Binghamton University, Binghamton, NY 13902, USA*

E-mail: lpiper@binghamton.edu

Supplementary Figures

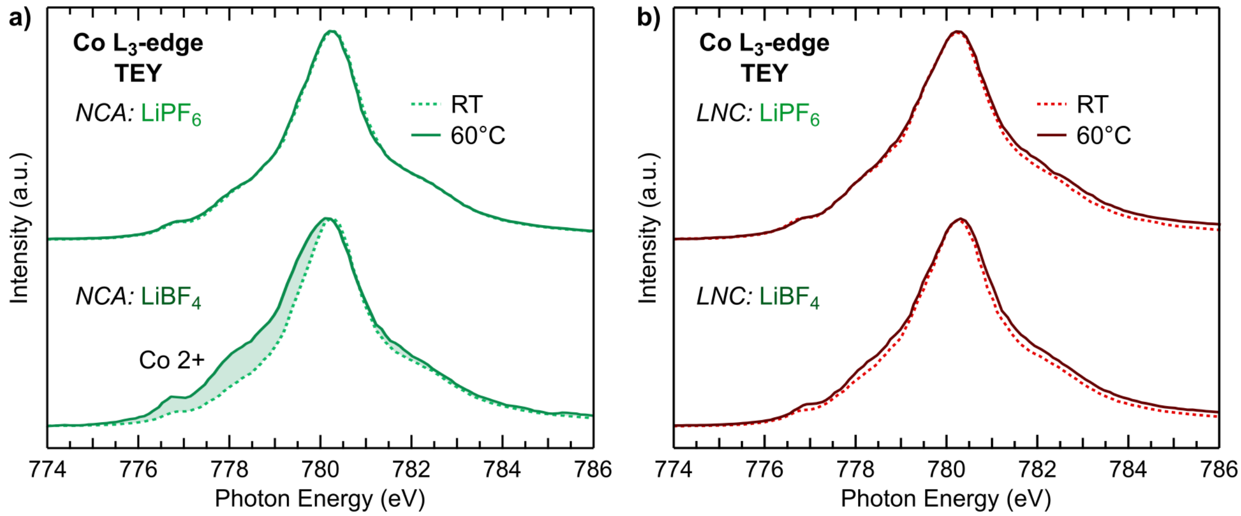

Figure S1: Co L_3-edge measurements for a) $LiNi_{0.8}Co_{0.15}Al_{0.05}O_2$ (NCA) and b) $LiNi_{0.8}Co_{0.2}O_2$ (LNC) electrodes charged to 4.75 V at room temperature (24 °C) with no constant voltage (referred to as RT electrode) and charged to 4.75 followed by a 10 hr constant voltage (CV) hold, all at 60 °C (referred to as 60 °C electrode). In the $LiPF_6$ case, the Co L_3-edge shows minimal change between the RT and 60 ° electrodes (spectra overlaps). This can be contrasted with the $LiBF_4$ case, where the NCA electrode shows a pronounced increase in lower energy features associated with cobalt surface reduction under thermal stress.

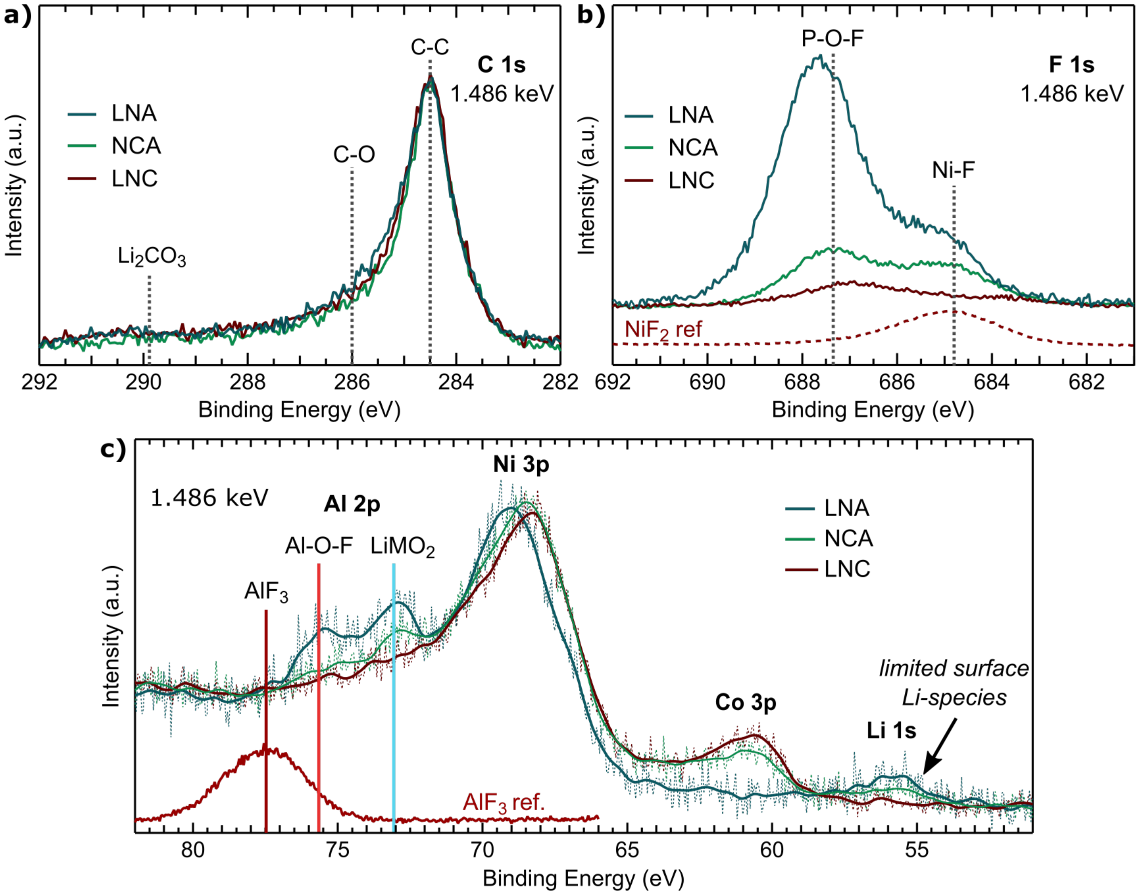

Figure S2: XPS measurements of the a) C 1s, b) F 1s, c) Al 2p, Ni 3p, Co 3p and Li 1s core region using Al Kα X-ray source (1.486 keV) for LNC, NCA, and $LiNi_{0.8}Al_{0.2}O_2$ (LNA) 60 °C electrodes. Normalizing the C 1s to the carbon black peak (C-C/284.5 eV), we find minimal difference lineshape related to the formation of new carbon environments related to electrolyte solvent decomposition. In the F 1s core region, the LNA 60 °C electrode shows the largest amount of fluorine surface species related to $LiPF_6$ decomposition. The higher binding energy peak at 687.7 eV is associated with P-O-F species while the lower binding energy peak at 685.3 may be associated with residual LiF and the formation of new Ni-F species. The binding energy region from 50 eV to 80 eV contains extensive information on the transition metal (TMs), aluminum, and lithium surface environments. Compared to the NCA and LNC 60 °C electrodes, LNA shows a shift in the Ni 3p core region to higher energy and the presence of a higher binding energy Al 2p peak related to the formation of Ni-F and Al-O-F species, respectively. In all three systems, there are minimal lithium surface species.

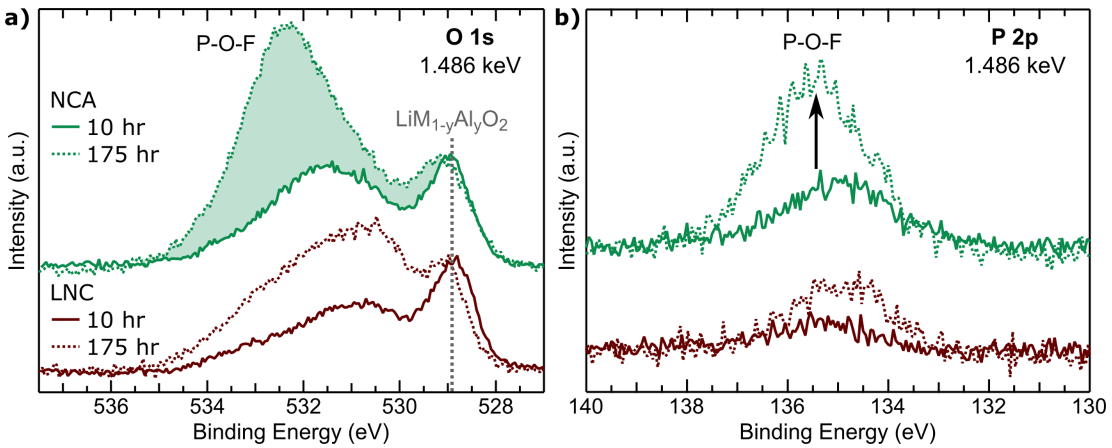

Figure S3: XPS measurements of the a) O 1s and b) P 2p core regions using Al Kα X-ray source (1.486 keV) for LNC and NCA electrodes held for 10 and 175 hr at 4.75 V and 60 °C. Longer holding results in more pronounced changes for the NCA system than the LNC system, where there is a pronounced buildup of P-O-F species at the surface (ndicated by shading in **a** and arrow in **b**).

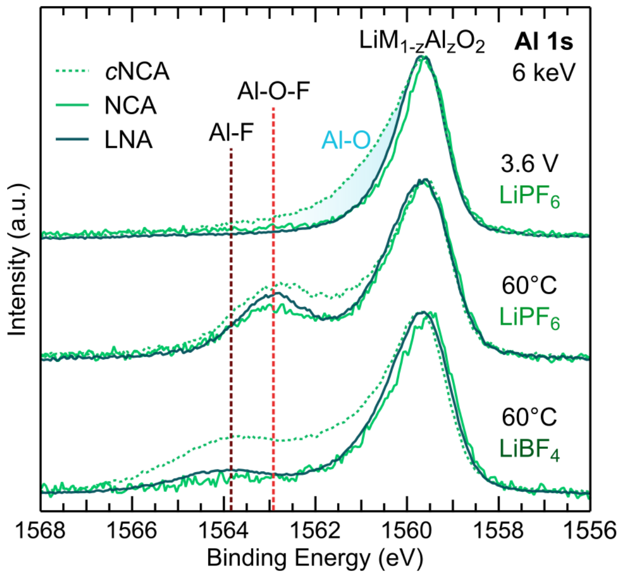

Figure S4: Al 1s core region for commercial NCA (cNCA) and in-house synthesized NCA and LNA electrodes charged to 3.6 V and 4.75 V at 60°C with a 10 hr CV hold using either $LiPF_6$ or $LiBF_4$. In the 3.6 V case, there is some variation in the asymmetry of the main Al 1s peak (1558-1560.5 eV) which is associated with differences in the amount of Al-O-like environments. For the 60 °C electrodes, we highlight the Al 1s peak at 1562.7 eV and 1564 when using $LiPF_6$ and $LiBF_4$, respectively. These peaks are assigned to Al-O-F-like environments and indicate an aluminum-electrolyte reaction that is dependent on the choice of electrolyte salt.

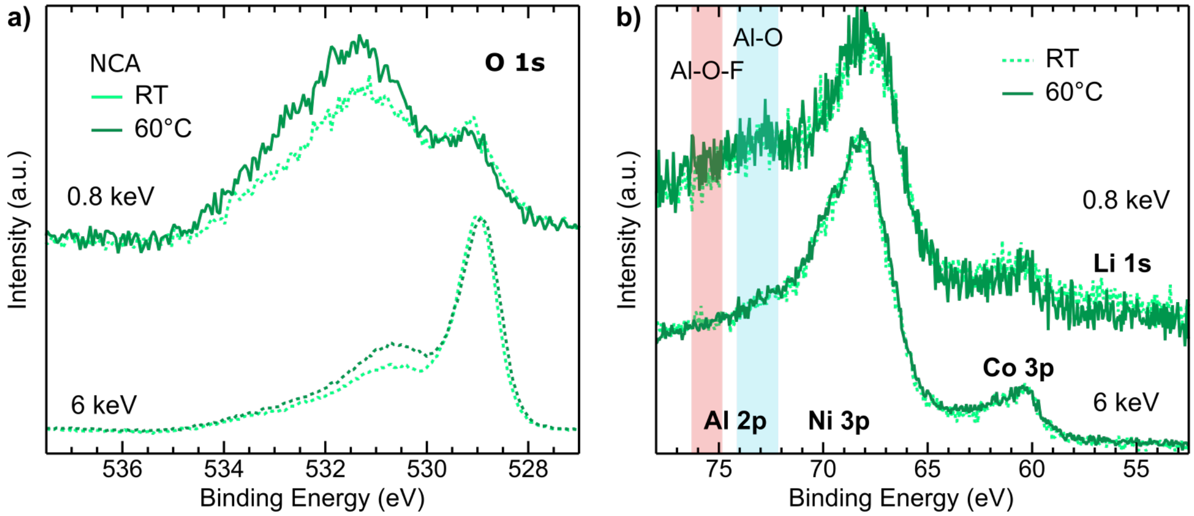

Figure S5: Depth-dependent XPS measurements at 0.8 and 6 keV of the a) O 1s and b) Al2p/Ni3p regions for NCA RT and 60°C electrodes. In the O 1s core region, there is only a slight increase in the surface peaks between the RT and 60 °C when using $LiPF_6$. This is much less pronounced than the increase in surface peaks observed for LNA presented in Fig. 6 of the main text. For NCA with only 5% Al-doping, spectral contamination from the Ni 3p leads to difficulty in using the Al 2p core region to examine aluminum surface and bulk environments. In the Ni 3p and Li 1s core regions, we find minimal difference between the RT and 60°C electrodes associated with the formation of new CEI species.

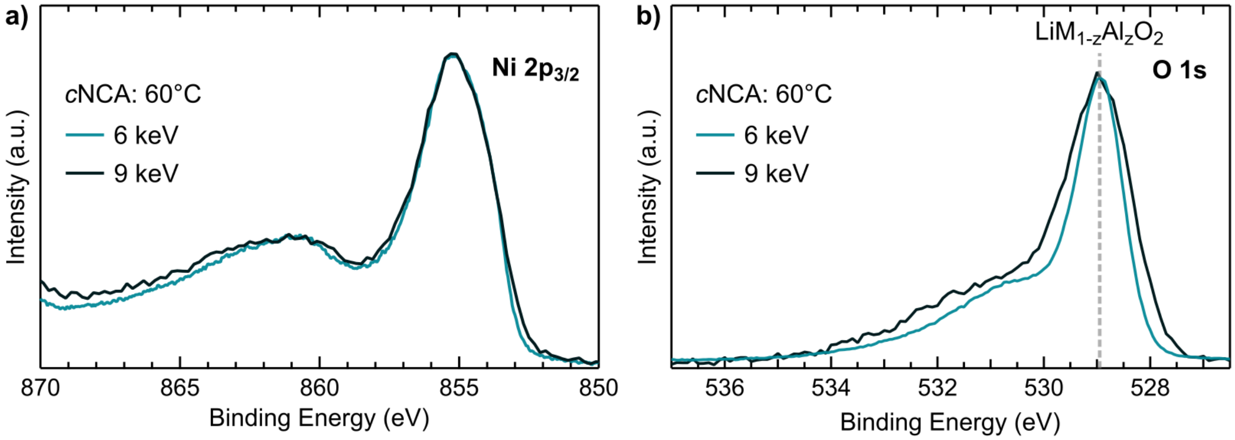

Figure S6: HAXPES measurements of the a) Ni $2p_{3/2}$ and b) O 1s core region using the 9 keV (9.25 keV) Ga Kα lab based X-ray source compared to 6 keV measurements collected at beamline I09 at the Diamond Light Source Ltd. Similar quality data for these oxide cathodes is obtained with the lab based HAXPES while providing probing depths beyond 30 nms.

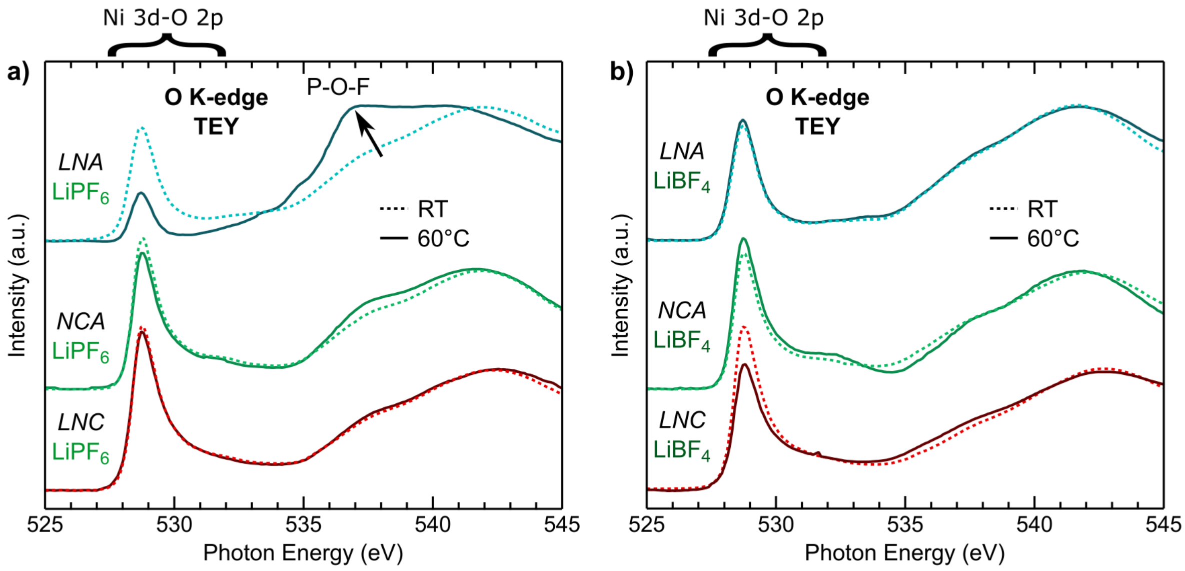

Figure S7: O K-edge spectra in surface sensitive total electron yield (TEY) mode for LNA, NCA, and LNC RT and 60°C electrodes using a) $LiPF_6$ and b) $LiBF_4$. In the $LiPF_6$ case, the LNA shows a pronounced drop in the Ni 3d-O 2p pre-edge peaks (527 to 533 eV) and a change in the higher energy lineshape associated with the formation of new P-O-F species. In the $LiBF_4$ case, there is limited change in any of the O K-edge lineshapes except for the LNC electrode that shows a decrease in the pre-edge peak as a result of the 60 °C testing conditions.

Supplementary Tables

Table S1: **Aluminum peak fit results** Peak fits were conducted on cNCA, NCA, and LNA electrodes with either $LiPF_6$ or $LiBF_4$ as the electrolyte salt using the following testing conditions: 1) charged to 3.6 V as a reference, 2) charged to 4.75 V at RT, 3) charged to 4.75 V at 60°C followed by a 10 hr CV hold, and 4) charged to 4.75 V at 60°C followed by a 175 hr CV hold. The last testing condition was only used for the *c*NCA case as using $LiPF_6$ under these conditions results in a complex exothermic reaction that may influence the cathode-electrolyte reactions.[S1]

Sample	$\mathbf{LiM_{1-z}Al_zO_2}$ ~1559.55 eV % Comp.	**Al-O 1** ~1560.35 eV % Comp.	**Al-O 2** ~1561.15 eV % Comp.	**Al-O-F** ~1562.7 eV % Comp.	**Al-F** ~1564 eV % Comp.
		***c*NCA using $LiPF_6$**			
Prist. Powd.	65%	16%	11%	8%*	-
3.6 V	61%	16%	12%	11%*	-
4.75 RT**	39%	33%	11%	17%	-
4.75 60°C+10hr**	44%	13%	14%	23%	6%
4.75 60°C+175hr	27%	18%	8%	37%	10%
		**NCA using $LiPF_6$**			
3.6 V	74%	15%	6%	5%*	-
4.75 V RT	61%	13%	13%	13%*	-
4.75 V 60°C+10hr	49%	18%	5%	28%	-
		**LNA using $LiPF_6$**			
3.6 V	71%	21%	5%	3%*	-
4.75 V RT	63%	20%	9%	8%*	-
4.75 V 60°C+10hr	44%	23%	5%	28%	-
		***c*NCA using $LiBF_4$**			
3.6 V	50%	19%	20%	11%*	-
4.75 V RT**	46%	17%	20%	17%	-
4.75 V 60°C+10hr**	37%	13%	16%	10%	24%
4.75 V 60°C+175hr	28%	14%	14%	7%	37%
		**NCA using $LiBF_4$**			
3.6 V	54%	34%	5%	7%*	-
4.75 V RT	59%	23%	9%	9%*	-
4.75 V 60°C+10hr	64%	16%	12%	-	8%
		**LNA using $LiPF_6$**			
3.6 V	66%	21%	8%	5%*	-
4.75 V RT	64%	21%	9%	6%*	-
4.75 V 60°C+10hr	49%	24%	10%	2%	15%

*May be partially associated with Al 1s satellite peak such as in *c*NCA prist. with no $LiPF_6$ contact

**Electrodes measured in discharged state (2.7 V)

References

(S1) Faenza, N. V.; Lebens-Higgins, Z. W.; Mukherjee, P.; Sallis, S.; Pereira, N.; Badway, F.; Halajko, A.; Ceder, G.; Cosandey, F.; Piper, L. F. J.; Amatucci, G. G. Electrolyte-Induced Surface Transformation and Transition-Metal Dissolution of Fully Delithiated $LiNi_{0.8}Co_{0.15}Al_{0.05}O_2$. *Langmuir* **2017**, *33*, 9333–9353.
